# Activation of Eosinophils Interacting with Bronchial Epithelial Cells by Antimicrobial Peptide LL-37: Implications in Allergic Asthma

**DOI:** 10.1038/s41598-017-02085-5

**Published:** 2017-05-12

**Authors:** Delong Jiao, Chun-Kwok Wong, Miranda Sin-Man Tsang, Ida Miu-Ting Chu, Dehua Liu, Jing Zhu, Man Chu, Christopher Wai-Kei Lam

**Affiliations:** 1 Department of Chemical Pathology, The Chinese University of Hong Kong, Prince of Wales Hospital, Shatin, NT Hong Kong China; 2 Institute of Chinese Medicine and State Key Laboratory of Phytochemistry and Plant Resources in West China, the Chinese University of Hong Kong, Hong Kong, China; 3 Shenzhen Research Institute, The Chinese University of Hong Kong, Shenzhen, China; 4 State Key Laboratory of Quality Research in Chinese Medicine, Macau Institute for Applied Research in Medicine and Health, Macau University of Science and Technology, Taipa, Macau China

## Abstract

The role of antimicrobial peptide LL-37 in asthma exacerbation is unclear. Microbial infection, which is the most common inducer of asthma exacerbation, is accompanied by elevated LL-37. The present study found that co-culture of eosinophils and bronchial epithelial cell line BEAS-2B significantly enhanced intercellular adhesion molecule-1 on both cells and CD18 expression on eosinophils upon LL-37 stimulation. IL-6, CXCL8 and CCL4 were substantially released in co-culture in the presence of LL-37. LL-37 triggered the activation of eosinophils interacting with BEAS-2B cells in a P2X purinoceptor 7/epidermal growth factor receptor-dependent manner. Eosinophils and BEAS-2B cells differentially contribute to the expression of cytokines/chemokines in co-culture, while soluble mediators were sufficient to mediate the intercellular interactions. Intracellular p38-mitogen-activated protein kinase, extracellular signal-regulated kinase and NF-κB signaling pathways were essential for LL-37-mediated activation of eosinophils and BEAS-2B cells. By using the ovalbumin-induced asthmatic model, intranasal administration of mCRAMP (mouse ortholog of LL-37) in combination with ovalbumin during the allergen challenge stage significantly enhanced airway hyperresponsiveness and airway inflammation in sensitized mice, thereby implicating a deteriorating role of LL-37 in allergic asthma. This study provides evidence of LL-37 in triggering asthma exacerbation via the activation of eosinophils interacting with bronchial epithelial cells in inflammatory airway.

## Introduction

The human innate immune system consists of a broad spectrum of cells and molecules for the defense against pathogens as well as the induction of inflammation^1^. Antimicrobial peptides, the evolutionarily conserved molecules employed by the host to eliminate microorganisms, possess multiple biological functions. Among different antimicrobial peptides, LL-37, a 37 amino acid cationic peptide generated by cleavage of the C terminal end of hCAP18 protein, is the only cathelicidin-family antimicrobial peptide that has been found in human^2^. Various stimulants, including bacteria, viruses, Vitamin D3 and short chain fatty acids have been reported to be inducers of LL-37 expression in innate cells such as macrophages, neutrophils and epithelial cells^3^. LL-37 signals via Formyl peptide receptor 2 (FPR2), P2X purinoceptor 7 (P2X7R), epidermal growth factor receptor (EGFR) and human epidermal growth factor receptor 2 (ERBb2), which are expressed on various inflammatory cells and epithelial cells^4–7^. Consequently, diverse biological functions including angiogenesis, wound healing, cell apoptosis and immunomodulation are induced by LL-37 beside its intrinsic antimicrobial activities^8^.

Asthma is an increasingly prevalent allergic disease that is characterized by T helper type 2 (Th2) lymphocyte dominated airway inflammation, hypersensitivity and remodeling^9^. Dysregulated LL-37 levels are found in the sputum of asthmatic patients^10^. So far, the role of LL-37 in the pathogenesis of allergic asthma remains unclear, though publications have demonstrated its pro-inflammatory role in regulating the allergic inflammation. LL-37 can directly function as a chemotactic factor to induce the recruitment of the effector cells in asthma such as neutrophils, eosinophils and mast cells^5, 11^. Indirectly, LL-37 induces the recruitment of neutrophils by mediating the asthma-related neutrophil chemokine CXCL8 expression in airway epithelial cells and smooth muscle cells^4, 12^. Recently LL-37 has been reported to induce the release of the proinflammatory, spasmogenic cysteinyl leukotrienes (CysLTs) from human eosinophils, implicating an immunopathological role of LL-37 in asthma by mediating the expression of CysLTs^13^. Asthma exacerbation is an aggravated disease stage in asthmatic patients that is triggered by diverse factors, including viral/bacterial infections and allergen/irritant exposures^14^. Microbial infections, one of the most common inducers of LL-37 release, account for ~50% of asthma exacerbations^15^. However, the exact role of LL-37 in asthma exacerbation remains to be elucidated.

Eosinophils have been reported to be associated with a variety of allergic disorders including asthma^16^. As the principal effector cells of allergic inflammation, eosinophils have long been accepted as the hallmark of Th2-type immune responses^17^. Activated eosinophils release cytotoxic granular proteins and inflammatory mediators, causing epidermal damage, tissue swelling, and recruitment of inflammatory cells^18^. Moreover, eosinophils have been shown to express receptors involved in LL-37 mediated signalling including FPR2 and P2X7R^13, 19^. Although previous studies have established a close link between respiratory viral/bacterial infections and asthma exacerbation^14^, it remains unknown whether the expression of LL-37 that associates with the innate response to airway infections might be involved in the exacerbation of the disease by activating eosinophils. Moreover, our previous investigations have found that the interaction between eosinophils and epithelial cells can result in an amplified stimulating effects induced by a variety of proinflammatory cytokines, hormone leptin and bacterial pathogen associated molecular patterns (PAMPs)^20–22^. Therefore, the aim of the present study was to investigate the underlying mechanisms between LL-37 and asthma exacerbation both *in vitro* by using co-culture system of eosinophils and bronchial epithelial cells and *in vivo* by using ovalbumin (OVA)-induced mouse model of allergic airway inflammation.

## Results

### Antimicrobial peptides LL-37 induced the expression of adhesion molecules and pro-inflammatory cytokines/chemokines in co-cultured eosinophils and BEAS-2B cells

**Figure 1:**
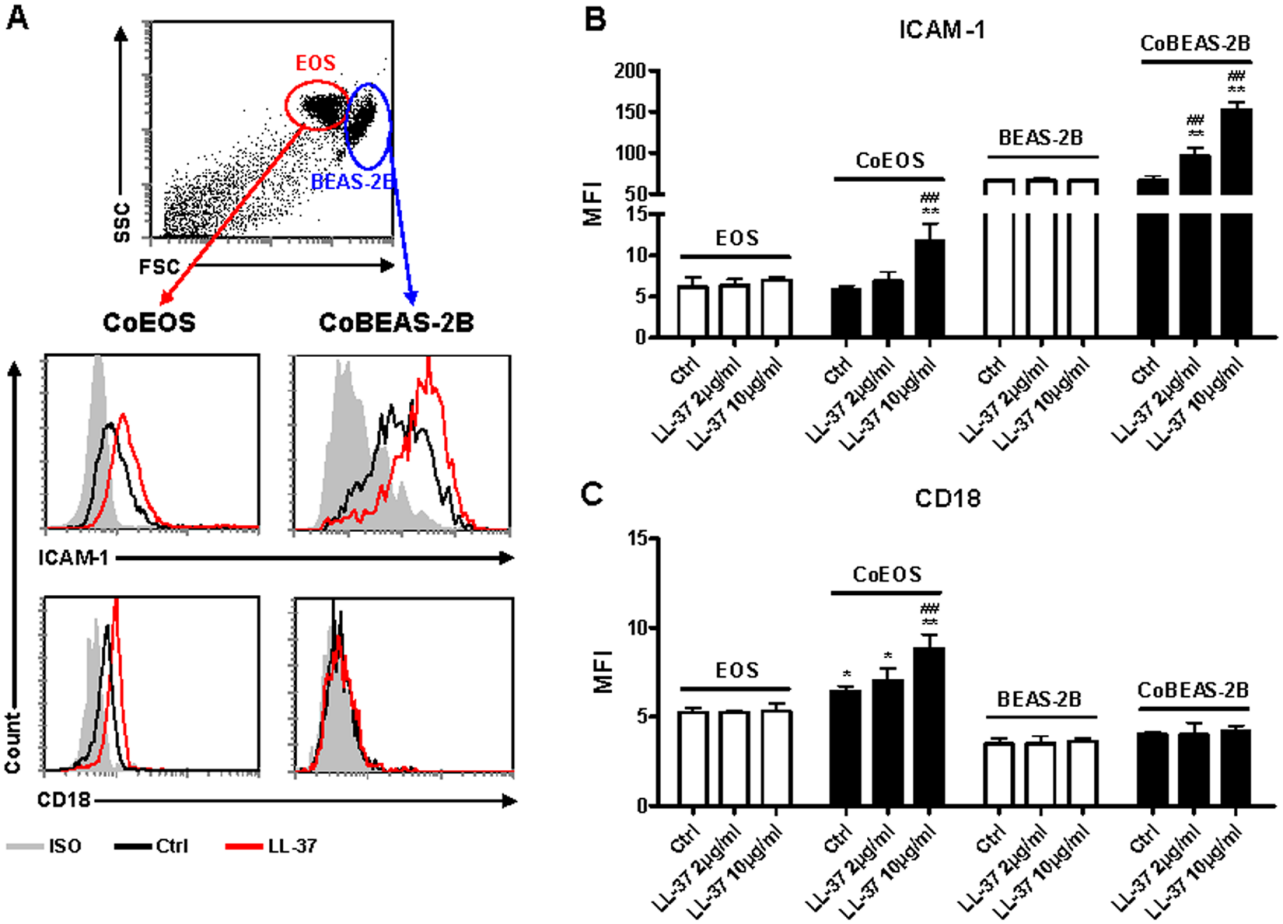
Effects of LL-37 on the surface expression of ICAM-1 and CD18 on eosinophils and BEAS-2B cells in the co-culture. Human eosinophils (3 × 10^5^) and BEAS-2B cells (1 × 10^5^) were cultured either together or separately with or without 2 or 10 μg/ml LL-37 for 20 h. (**A**) representative dot plot and histograms illustrating differential flow cytometric analysis of surface expression of ICAM-1 and CD18 on eosinophils or BEAS-2B cells in the co-culture of triplicate independent experiments showing essentially similar results. (**B,C**) quantitative results of surface ICAM-1 (**B**) or CD18 (**C**) expression on eosinophils and BEAS-2B cells are presented as MFI. EOS, eosinophils; CoEOS, eosinophils in co-culture; CoBEAS-2B, BEAS-2B cells in co-culture. Results are shown as arithmetic mean plus SD of triplicate independent experiments. *P < 0.05, **P < *0.01* when compared between denoted groups and eosinophil/fibroblast control groups. ^*##*^ *P* < *0.01* when compared between denoted groups and co-culture control groups.

**Figure 2:**
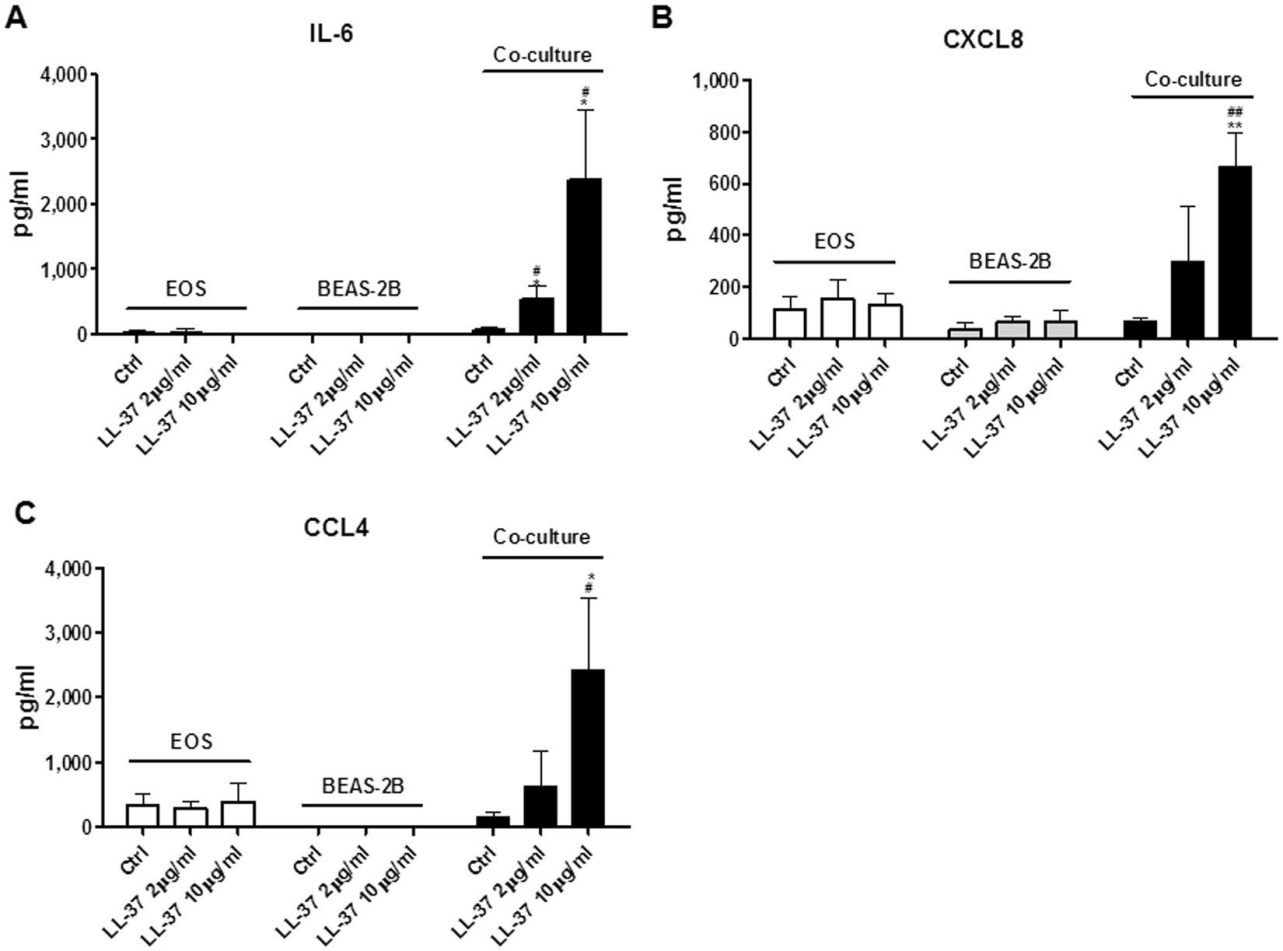
Effects of LL-37 on the induction of cytokines/chemokines upon co-culture of eosinophils and BEAS-2B cells. Human eosinophils (3 × 10^5^) and BEAS-2B cells (1 × 10^5^) were cultured either together or separately with or without 10 μg/ml LL-37 for 20 h. Release of IL-6 (**A**), CXCL8 (**B**) and CCL4 (**C**) in culture supernatant was determined. EOS, eosinophils. Results are shown as arithmetic mean plus SD of triplicate independent experiments. *P < 0.05, **P < *0.01* when compared between denoted groups and eosinophil control groups. ^*#*^ *P* < *0.05*, ^*##*^ *P* < *0.01* when compared between denoted groups and co-culture control groups.

Previous studies have established the role of LL-37 in modulating eosinophilic airway inflammation through chemotaxis and CysLTs release^5, 13^, while the direct stimulating effects of LL-37 on human eosinophils remained unclear. Therefore, we investigated whether LL-37 might activate human eosinophils and up-regulate the expression of adhesion molecules and asthma-related cytokines/chemokines. As shown in Figs 1B,C and 2A–C, the addition of LL-37 (2 and 10 µg/ml) in eosinophil cultures did not affect the surface expression of adhesion molecules ICAM-1/CD18 or the release of asthma-related inflammatory IL-6, CXCL8 and CCL4. Furthermore, higher concentrations of LL-37 (50 µg/ml) exhibited no significant effects on the expression of adhesion molecules or cytokines/chemokines in eosinophil cultures (all P > 0.05, data not shown).

We next hypothesized that intercellular interaction between bronchial epithelial cells and eosinophils might enhance the stimulating effects of LL-37. To test this hypothesis, BEAS-2B cells, a widely used human bronchial epithelial cell line, were co-cultured with eosinophils before stimulation with increasing concentrations of LL-37. BEAS-2B cells alone did not respond to LL-37 stimulation judging from the unaffected expression levels of ICAM-1/CD18, IL-6, CXCL8 and CCL4 (Figs 1B,C and 2A–C). Strikingly, significantly increased surface expressions of ICAM-1 were found on both eosinophils and BEAS-2B cells in co-culture in the presence of 10 µg/ml LL-37, while LL-37 (2 µg/ml) promoted ICAM-1 expression on co-cultured BEAS-2B cells (Fig. 1A,B). Although no variations in CD18 were observed on BEAS-2B cells, significantly higher levels of surface CD18 expression were detected on co-cultured eosinophils with LL-37 (10 µg/ml) stimulation (Fig. 1A,C). Moreover, the co-culture of eosinophils and BEAS-2B cells released larger amount of IL-6, CXCL8 and CCL4 in the presence of LL-37 (Fig. 2A–C). These findings collectively indicate that intercellular interactions between eosinophils and bronchial epithelial cells are necessary for the optimal LL-37-mediated cell activation.

Using the same experimental condition, similar ICAM-1 induction in co-culture of human eosinophils and primary human bronchial epithelial cells (Cell Applications, Inc., CA, USA) was observed upon LL-37 (10 μg/ml) stimulation, comparing with that using BEAS-2B cell line in Fig. 1B (MFI of ICAM-1 expression on primary bronchial epithelial cells: 51.4 ± 4.0 and 85.8 ± 0.3, without and with LL-37 activation, respectively; and eosinophils: 74.4 ± 1.2 and 90.0 ± 1.6, without and with LL-37 activation, respectively, both p < 0.01). The above results therefore confirm the similar biological responses between BEAS-2B cells and primary human bronchial epithelial cells upon LL-37 activation.

### P2X7R and EGFR mediate LL-37-induced activation of eosinophils interacting with BEAS-2B cells

**Figure 3:**
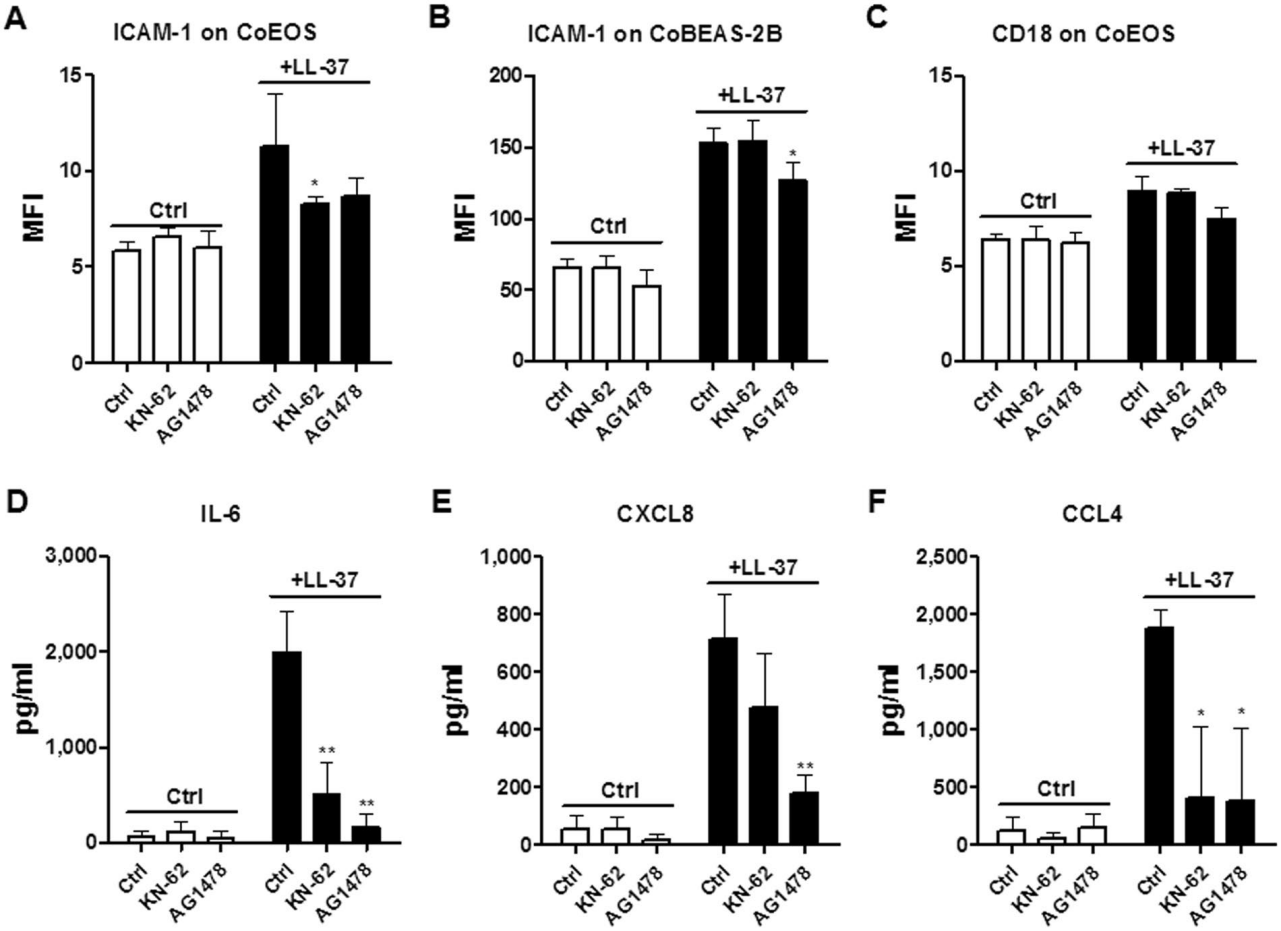
Effects of KN-62 and AG1478 on the expression of adhesion molecules and cytokines/chemokines in co-cultured eosinophils and BEAS-2B cells under LL-37 stimulation. Human eosinophils (3 × 10^5^) co-cultured with BEAS-2B cells (1 × 10^5^) were pretreated with KN-62 (5 µM) and AG1478 (1 µM) for 1 hour, followed by incubation with 10 µg/ml LL-37 for further 20 h. ICAM-1 expression on eosinophils (**A**), BEAS-2B cells (**B**), and the CD18 expression on eosinophils (**C**) were analyzed and expressed as previously described. Release of IL-6 (**D**), CXCL8 (**E**) and CCL4 (**F**) in culture supernatant was determined. Results are shown as arithmetic mean plus SD of triplicate independent experiments. *P < 0.05, **P < *0.01* when compared between the denoted groups and the corresponding control groups with LL-37 treatment.

Diverse receptors, including FPR2, P2X7R, EGFR and ERBb2 have been reported to mediate a variety of cellular responses induced by LL-37^3^. To examine which receptors are indispensible for the LL-37-triggered activation of eosinophils interacting with BEAS-2B cells, small molecule inhibitors were added to the co-culture system prior to LL-37 stimulation. As shown in Fig. 3A,B, in the eosinophil/BEAS-2B cell co-culture, P2X7R inhibitor KN-62 significantly suppressed LL-37-induced ICAM-1 expression on eosinophils, while EGFR inhibitor AG1478 significantly suppressed ICAM-1 expression on BEAS-2B cells. Although not statistically significant, EGFR inhibitor AG1478 also inhibited the expression of CD18 on co-cultured eosinophils (Fig. 3C). The LL-37-induced release of IL-6 and CCL4 were significantly suppressed by both KN-62 and AG1478 in eosinophil-BEAS-2B cell co-culture, whereas CXCL8 induction was inhibited only by AG1478 (Fig. 3D–F). However, neither the expression of ICAM-1/CD18 nor the release of cytokines/chemokines were suppressed by FPR2 antagonist WRW4 or ERBb2 antagonist AG825 in co-cultured eosinophils and BEAS-2B cells under LL-37 stimulation (data not shown). The above findings suggested that P2X7R and EGFR were essential for LL-37-mediated activation of eosinophils and BEAS-2B cells. However, whether FPR2 and ERBb2 are involved in the activation of eosinophils and BEAS-2B cells may need further investigations. We also found that WRW4 and LL-37 could suppress and up-regulate the expression level of phosphorylated ERK level in eosinophils, respectively, while WRW4 priming could also suppress LL-37-induced ERK phosphorylation in eosinophils (data not shown). Our results are therefore compatible with those of a previous study to verify the suppressing activity of our WRW4 on FPR2^13^.

### Sources of cytokines/chemokines release in the co-culture systems upon LL-37 stimulation

**Figure 4:**
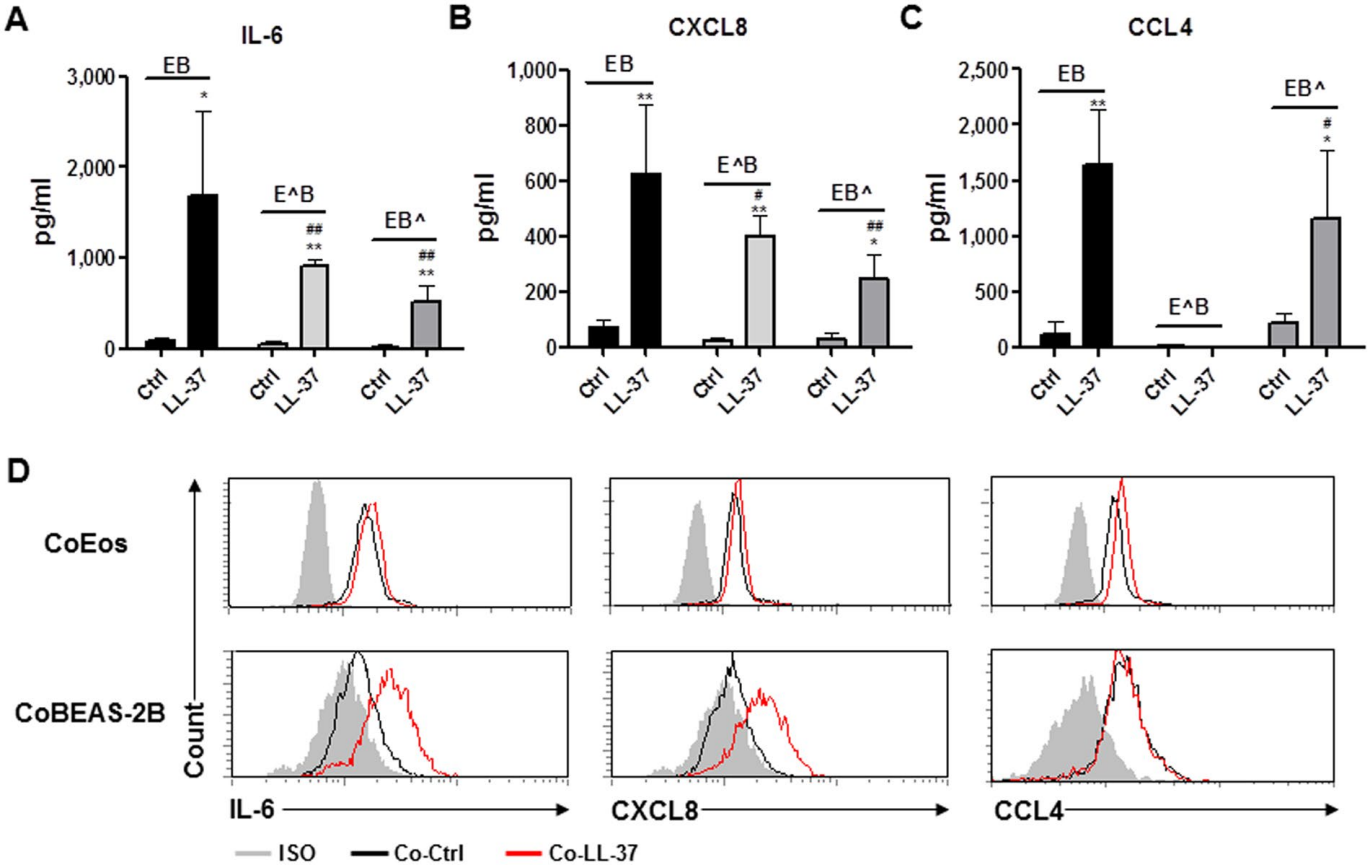
Sources of cytokine/chemokine expressions in co-culture of eosinophils and BEAS-2B cells under the stimulation of LL-37. Human eosinophils (3 × 10^5^) and BEAS-2B cells (1 × 10^5^) were treated with or without paraformaldehyde before being cultured together with or without 10 µg/ml LL-37 for 20 h. Release of IL-6 (**A**), CXCL8 (**B**) and CCL4 (**C**) in culture supernatant was determined. (**D**) 3 × 10^5^ eosinophils and 1 × 10^5^ BEAS-2B cells were co-cultured with or without 10 µg/ml LL-37 for 16 h, followed by the incubation with 10 µg/ml brefeldin A for further 4 h. Intracellular staining of IL-6, CXCL8 and CCL4 were then performed. Representative histograms of intracellular staining of IL-6, CXCL8 and CCL4 are shown from triplicate independent experiments showing essentially similar results. E: unfixed eosinophils; E^: fixed eosinophils; B: unfixed BEAS-2B cells; B^: fixed BEAS-2B cells. Results are shown as arithmetic mean plus SD of triplicate independent experiments. *P < 0.05, **P < *0.01* when compared between the denoted group and the corresponding control groups without treatment. ^*#*^ *P* < *0.05*, ^*##*^ *P* < *0.01* when compared between the denoted groups and the corresponding control groups of unfixed cells with LL-37 treatments.

To investigate the source of cytokines/chemokines released in the co-culture system, we compared the levels of cytokine/chemokine release between normal intact cells and paraformaldehyde-fixed cells in co-culture. In the eosinophil-BEAS-2B cell co-culture, the release of IL-6 and CXCL8 were significantly suppressed by the fixation of either eosinophils or BEAS-2B cells (Fig. 4A,B). In contrast, CCL4 production was completely abrogated upon the fixation of eosinophils alone, but not BEAS-2B cells (Fig. 4C). These results indicate that both eosinophils and BEAS-2B cells contributed to LL-37-mediated release of IL-6 and CXCL8 in co-culture, whereas co-cultured eosinophils were the main source of LL-37-induced CCL4 expression.

To confirm the expression of cytokines/chemokines in LL-37-stimulated eosinophils and/or BEAS-2B cells, intracellular staining of IL-6, CXCL8 and CCL4 proteins were preformed after 4 h incubation with the protein transport inhibitor brefeldin A. As shown in Fig. 4D, increased intracellular expressions of IL-6 and CXCL8 were found in both eosinophils and BEAS-2B cells in co-culture upon the stimulation of LL-37. However, only co-cultured eosinophils were found to express higher level of CCL4 in the presence of LL-37 (Fig. 4D). Consistently, these data suggest that in co-culture, LL-37 triggered the release of IL-6, CXCL8 and CCL4 in eosinophils as well as the expression of IL-6 and CXCL8 in BEAS-2B cells.

### Effects of transwell insert on cytokine/chemokine release in the co-culture systems upon stimulation with LL-37

**Figure 5:**
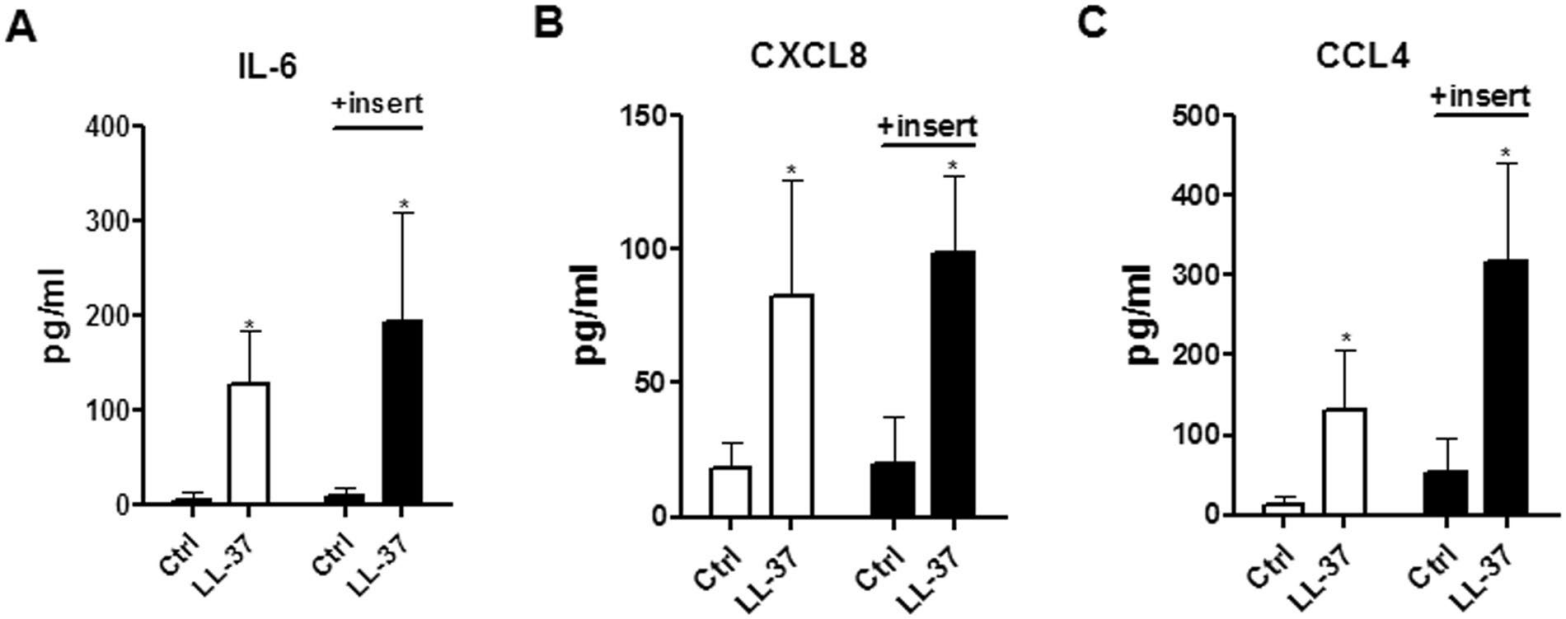
Effects of transwell inserts on cytokine/chemokine expressions in co-culture of eosinophils and BEAS-2B cells upon the stimulation of LL-37. Human eosinophils (3 × 10^5^) and BEAS-2B cells (1 × 10^5^) were cultured together with or without 10 µg/ml LL-37 in the presence or absence of transwell inserts for 20 h. Release of IL-6 (**A**), CXCL8 (**B**) and CCL4 (**C**) in culture supernatant was determined. Results are shown as arithmetic mean plus SD of triplicate independent experiments. **P < *0.01* when compared between the denoted group and the corresponding control groups without treatment.

To explore whether the direct intercellular contact was essential for the induction of cytokines/chemokines in the co-culture of eosinophils and BEAS-2B cells, transwell inserts with pore size of 0.4 μm were used to separate the co-cultured cells into two compartments. Intercellular communication by soluble mediators, rather than direct intercellular interaction, was allowed in this co-culture system with transwell insertions. As shown in Fig. 5A–C, the separation of eosinophils and BEAS-2B cells in co-culture by transwell inserts failed to affect LL-37-mediated expression of IL-6, CXCL8 and CCL4. Therefore, soluble mediators, rather than direct intercellular contact, accounted for LL-37-mediated cell activation in co-culture systems.

### Signaling transductions via NF-κB, p38-MAPK and ERK pathways were responsible for the activation of eosinophils and BEAS-2B cells in response to LL-37 stimulation

**Figure 6:**
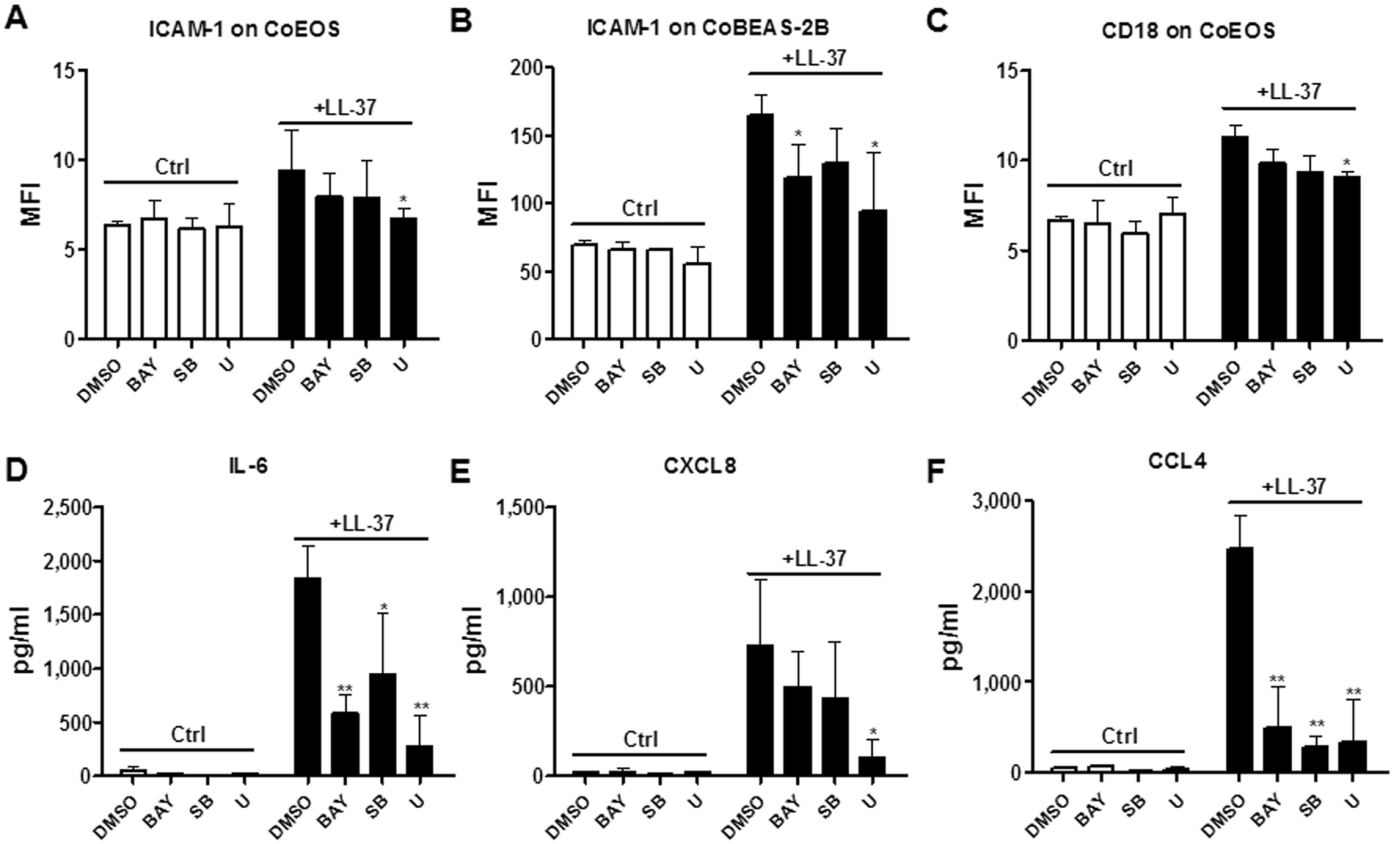
Inhibitors for p38-MAPK, NF-κB and ERK-MAPK pathways abolished LL-37-induced activation of human eosinophils co-cultured with BEAS-2B cells. Human eosinophils (3 × 10^5^) co-cultured with BEAS-2B cells (1 × 10^5^) were pretreated with 7.5 μM SB203580 (p38-MAPK inhibitor), 1 μM BAY11-7082 (NF-κB inhibitor), 10 μM U0126 (ERK inhibitor) for 1 h, followed by incubation with 10 µg/ml LL-37 for further 20 h. ICAM-1 expression on eosinophils (**A**) and BEAS-2B cells (**B**) as well as CD18 expression on eosinophils (**C**) was analyzed and expressed as previously described. Release of IL-6 (**D**), CXCL8 (**E**) and CCL4 (**F**) in the culture supernatant was determined. BAY, BAY11-7082; SB, SB203580; U, U0126. Results are shown as arithmetic mean plus SD of triplicate independent experiments. *P < 0.05, **P < *0.01* when compared between the denoted groups and the corresponding control groups with LL-37 treatments. DMSO was served as solvent control.

The activation of P2X7 has been extensively reported to induce the IL-1β and IL-6 expression through nuclear factor kappa-light-chain-enhancer of activated B cells (NF-κB), p38-mitogen-activated protein kinase (p38-MAPK) and extracellular signal-regulated kinase (ERK) pathways^23, 24^, while EGFR signal is one of the most common inducers of MAPK activation^25^. Therefore, we next focused our investigation on LL-37-mediated eosinophil/bronchial epithelial cell activation of P2X7/EGFR-induced intracellular signaling, including NF-κB and MAPK pathways. Firstly, we adopted a previously reported approach to screen for the potential signaling pathways involved in the activation of these cells by using small molecule inhibitors^26^. As shown in Fig. 6A–C, NF-κB inhibitor BAY11-7082 and ERK inhibitor U0126 differentially suppressed LL-37-induced expression of ICAM-1 and CD18 on eosinophils in co-culture, as well as ICAM-1 on BEAS-2B cells. Consistently, the release of IL-6, CXCL8 and CCL4 in eosinophils co-cultured with BEAS-2B cells were inhibited by SB203580, BAY11-7082 and U0126 (Fig. 6D–F). However, neither the increased ICAM-1/CD18 expressions nor the release of cytokines/chemokines were suppressed by c-Jun N-terminal kinases (JNK) inhibitor SP600125 and phosphoinositide-3-kinase–protein kinase B (PI3K-AKT) inhibitor LY294002 in eosinophils co-cultured with BEAS-2B cells under LL-37 stimulation (data not shown). These results suggested that, instead of the other signaling pathways, p38-MAPK, NF-κB and ERK were essential for LL-37-mediated activation of eosinophils and BEAS-2B cells.

**Figure 7:**
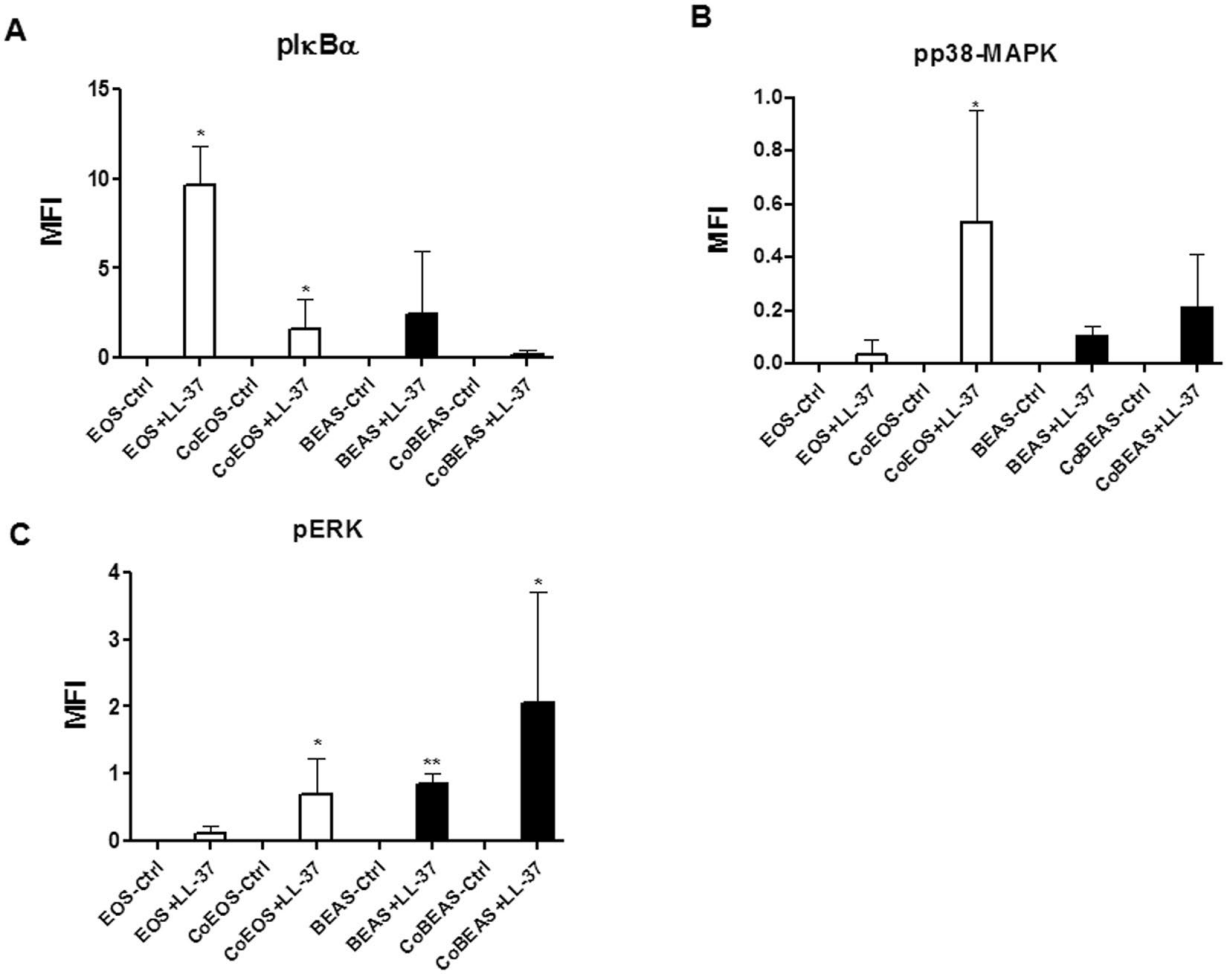
Activation of IκBα, p38-MAPK and ERK in human eosinophils co-cultured with BEAS-2B cells under LL-37 stimulation. Human eosinophils (1.2 × 10^6^) and BEAS-2B cells (4 × 10^5^) were cultured either together or separately with or without 10 µg/ml LL-37 for 10 minutes prior to fixation and permeabilization. Intracellular levels of phosphorylated IκBα (**A**), phosphorylated p38 MAPK (**B**), and phosphorylated ERK (**C**) in cells were measured by intracellular staining with specific antibodies and analyzed using flow cytometry. EOS, eosinophils; CoEOS, eosinophils in co-culture; BEAS, BEAS-2B cells; CoBEAS, BEAS-2B cells in co-culture. Results are expressed as MFI subtracting corresponding control groups and shown as arithmetic mean plus SD of triplicate independent experiments. *P < 0.05, **P < *0.01* when compared between the denoted groups and eosinophil or BEAS-2B cell control groups.

To further validate the activation of key signaling pathways induced by LL-37, intracellular staining of signaling molecules by quantitative flow cytometry was preformed. Figure 7A–C show that upon LL-37 stimulation in co-culture, intracellular IκBα, p38-MAPK and ERK pathways were differentially activated in eosinophils and BEAS-2B cells. Eosinophils expressed elevated levels of phosphorylated IκBα, p38-MAPK and ERK upon LL-37 treatment (Fig. 7A–C). The co-culture of eosinophils with BEAS-2B cells further enhanced LL-37-mediated p38-MAPK/ERK phosphorylation in eosinophils (Fig. 7B,C), though co-culture *per se* suppressed LL-37-induced IκBα phosphorylation in eosinophils (Fig. 7A). Moreover, BEAS-2B cells expressed higher levels of phosphorylated IκBα, p38-MAPK and ERK upon LL-37 treatment and co-culture further enhanced ERK phosphorylation in these cells (Fig. 7A–C).

### *In vivo* effect of mCRAMP on OVA-induced allergic airway inflammation

**Figure 8:**
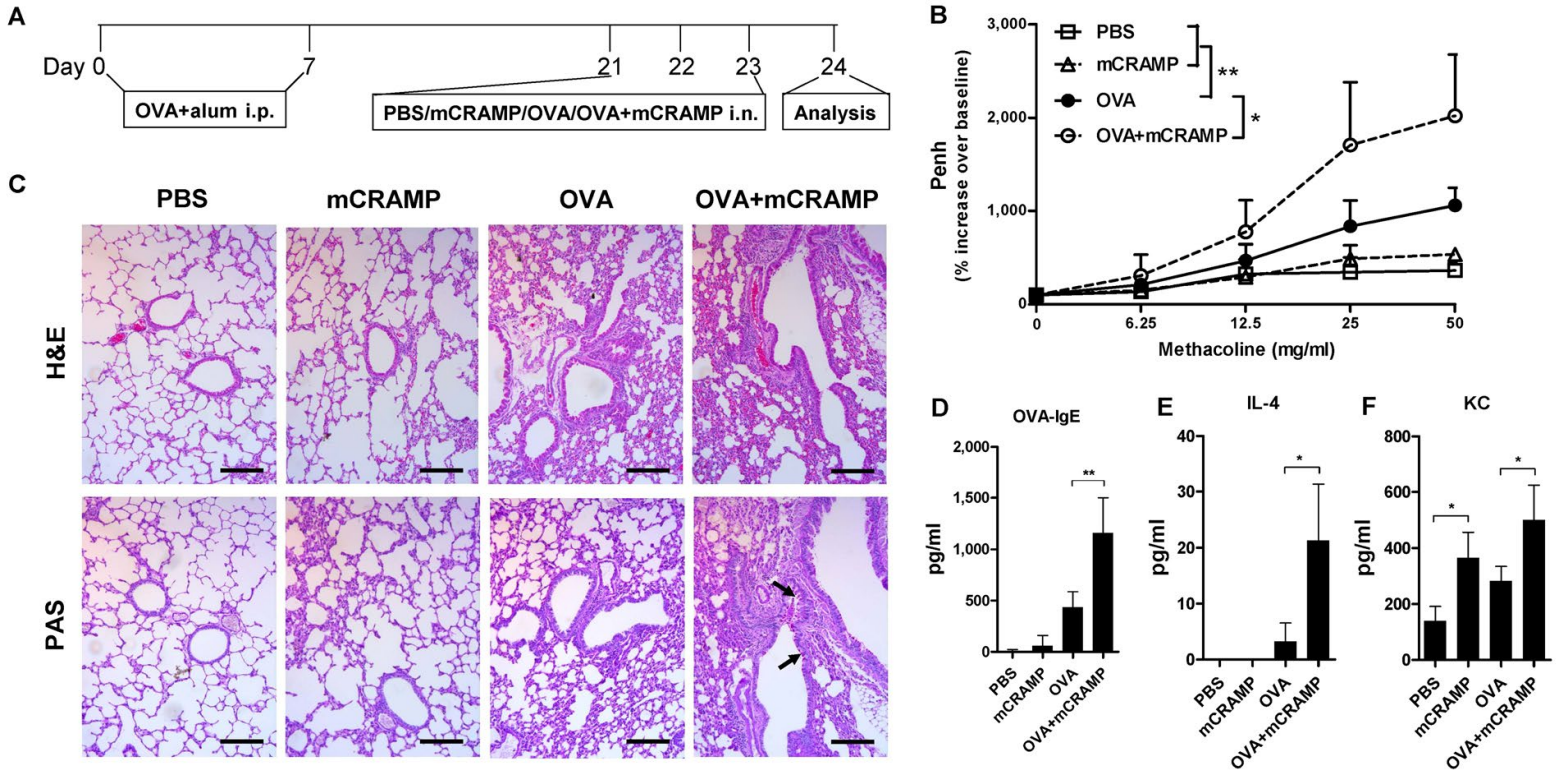
Intranasal administration of mCRAMP induced the exacerbation of OVA-induced airway hyperresponsiveness. (**A**) timeline protocol (Day 0 to Day 24) of OVA-induced allergic airway inflammation and intranasal mCRAMP administration. (**B**) increasing doses of inhaled methacholine-induced airway obstruction were presented as Penh (% increase over baseline), which was calculated by dividing the Penh value of different doses of methacholine by the Penh value of PBS baseline. (**C**) representative H&E and PAS staining of lung sections (x100 magnification). Black arrows denote bright purple-stained mucin-producing goblet cells. Scale bars = 100 µm. The concentrations of serum OVA-specific IgE (**D**) as well as IL-4 (**E**) and KC (**F**) in the lung homogenates were determined. *P < 0.05, **P < *0.01*. Data are representative from 2 independent experiments showing essentially similar results. Mice (n = 5) were used within each group for different treatments.

We found that *in vitro* stimulation with LL-37 could induce the activation of eosinophils by interacting with bronchial epithelial cells. Importantly, both the airway infiltration of eosinophils and the eosinophil-epithelial cell interactions are prominent features of the asthmatic airway inflammation. To explore whether LL-37 might trigger the exacerbation of allergic asthma *in vivo*, we took advantage of a well-established mouse model of asthmatic airway inflammation induced by OVA sensitization and challenge. By intranasal administration of mouse LL-37 ortholog-mCRAMP in combination with allergen OVA during the challenge phase of the model (Fig. 8A), the *in vivo* effects of mCRAMP on airway hypersensitivity (AHR) and airway inflammation were examined. Unlike PBS challenge, the intranasal OVA significantly enhanced airway hyperresponsiveness (Fig. 8B), inflammatory cell infiltration and mucus secretion (Fig. 8C) in sensitized mice. Although intranasal mCRAMP alone did not significantly affect AHR and airway inflammation, OVA-sensitized mice treated with OVA together with mCRAMP displayed a further increase in AHR and mucus secretion than OVA alone challenge (Fig. 8B,C). Moreover, mice that were challenged with OVA plus mCRAMP induced higher levels of serum OVA-specific IgE and lung homogenate IL-4/keratinocyte-derived cytokine (KC) in comparison with OVA-treated mice (Fig. 8D–F). Collectively, these results implicated a deteriorating role of LL-37 in allergic airway inflammation.

## Discussion

Despite diverse immunoregulatory functions reported for the antimicrobial peptide LL-37, its role in mediating the activation of human eosinophils has not been clearly demonstrated. Prerequisites of LL-37 expression such as respiratory infections/inflammations, are commonly observed in patients with allergic asthma, and have been documented as the major inducer for asthma exacerbations^14^. However, whether the elevated levels of LL-37 contribute to the aggravation of asthma is still unknown, and there have been no publication that elucidates the cellular and molecular pathways by which LL-37 leads to the activation of eosinophils. Herein, by using *in vitro* co-culture systems and *in vivo* asthmatic mouse model, we have demonstrated that LL-37 could trigger the activation of eosinophils upon interacting with bronchial epithelial cells and induce the exacerbation of allergic airway inflammation.

Previous studies have implied that LL-37 may induce the accentuation of allergic asthma by recruiting eosinophils into the inflammatory airway and triggering the release of CysLTs from eosinophils^5, 13^. However, the direct stimulating effect of LL-37 on human eosinophils remains to be addressed. Concentrations up to 50 µg/ml LL-37 have been used for achieving its biological functions such as antimicrobial activity and immunomodulation^8^. Therefore, the effects of pathophysiologically relevant concentrations of LL-37 (2, 10 and 50 µg/ml) on *in vitro* eosinophil culture system were examined. Although LL-37 (30 µg/ml) has been reported to induce chemotaxis of eosinophils and CysLTs release^5, 13^, in our experiments, LL-37 (2–50 µg/ml) failed to induce the expression of adhesion molecules and asthma-related cytokines/chemokines in human eosinophils (Figs 1B,C and 2A–C).

Predominant airway infiltration of eosinophils is one of the common characteristics of allergic asthma^16^. Histological analysis of the lungs of asthma patients reveals the proliferation of epithelial cells as well as dramatic infiltrations of eosinophils in bronchioles and small airways^27^. Being accessible to each other *in vivo*, it was hypothesized that eosinophils might be activated in synergy with bronchial epithelial cells in response to LL-37 stimulation. The BEAS-2B cells have been commonly used in many laboratories to substitute for primary bronchial epithelial cells because of the high proliferation rate and their independence on additional growth factors^28, 29^. Our previous studies have shown consistency between BEAS-2B and primary human bronchial epithelial cells in terms of the expression of cell surface adhesion molecules and the release of inflammatory cytokines/chemokines upon the stimulation of IL-17A and muramyl dipeptide^30, 31^. In the present study, we validated the stimulating effect of LL-37 (10 µg/ml) on eosinophils in co-culture with BEAS-2B cells, and results showed an enhanced expression of ICAM-1/CD18 as well as an increased release of pro-inflammatory cytokines/chemokines in co-culture in comparison to eosinophils or BEAS-2B alone cell cultures (Figs 1 and 2). These findings therefore suggest that the intercellular interactions between eosinophils and bronchial epithelial cells are prerequisites for the LL-37-induced cell activation.

Diverse receptors, including FPR2, P2X7R, EGFR and ERBb2, have been reported to transduce different LL-37-triggered cell signals and responses^3^. FPR2 is responsible for LL-37-mediated chemotaxis and CysLTs synthesis^5, 13^. P2X7R is accountable for LL-37-mediated IL-1β expression^32^. EGFR is necessary for LL-37-triggered CXCL8 release^4^. ERBb2 is required for LL-37-induced metastatic cancer phenotype^7^. Eosinophils have been shown to express LL-37 receptors including FPR2 and P2X7R^13,19^, while BEAS-2B cells constitutively express EGFR^33^. The expression of P2X7R on BEAS-2B cells has been elusive by now, but some studies have revealed the expression of P2X7R on airway epithelial cell line E10 and intestinal epithelial cells^34, 35^. By using small molecule inhibitors specific to these receptors, we identified the necessity of P2X7R and EGFR in LL-37-induced expression of adhesion molecules and cytokines/chemokines in co-cultured eosinophils and BEAS-2B cells (Fig. 3). These results, together with previous publications, further implicate an essential role of P2X7R/EGFR in LL-37-triggered *de novo* cytokine expression.

By fixing either eosinophils or BEAS-2B cells in the co-culture systems, we identified that upon LL-37 stimulation, both eosinophils and BEAS-2B cells were accountable for the release of IL-6 and CXCL8, whereas CCL4 was mainly produced by eosinophils (Fig. 4A–C). The intracellular staining of cytokines/chemokines in eosinophils and BEAS-2B cells in the co-culture system further validated these results (Fig. 4D). The incomplete coincidence of the expression of cytokines/chemokines between LL-37-activated eosinophils and BEAS-2B cells may be partly due to the distinct cytokine/chemokine transcriptome upon the same stimulus by different cells^36, 37^. Moreover, the results of experiments using transwell insertion showed that LL-37-mediated release of IL-6, CXCL8 and CCL4 were independent of direct intercellular contact between eosinophils and BEAS-2B cells (Fig. 5), suggesting a pivotal role of soluble mediators for the intercellular interactions and activation of cells. As phosphorylated-signaling molecules were rapidly up-regulated in co-cultured cells in comparison to cells in single culture within 10 minutes upon LL-37 treatment (Fig. 7A–C), we propose that these soluble mediators were induced by co-culture *per se*, rather than LL-37 stimulation. The soluble factors can be microvesicles/exosomes, cytokines/chemokines, lipids and reactive oxygen species that not only have pro-inflammatory functions, but also can freely transmit through the 0.4 μm transwell pore. These mediators should have no stimulating effects *per se*, they activate the cells in synergy with LL-37 stimulation. This hypothesis can be supported by the fact that significantly increased phosphorylated-signaling molecules were induced in co-cultured cells in comparison to cells in single culture within 10 minutes upon the treatment of LL-37 (Fig. 7A–C). Further experiments are on-going for the identification of such soluble factors.

Regarding the intracellular signaling mechanisms, previous reports have characterized the signal transduction pathways of LL-37-mediated eosinophils chemotaxis and CysLTs release^5, 13^, highlighting an important role of MAPK pathways in the pathogenesis of allergic asthma. By using optimal concentrations of small molecule inhibitors SB203580 (7.5 μM), BAY11-7082 (1 μM), U0126 (10 μM), LY294002 (5 μM) and SP600125 (3 μM) with maximum inhibitory effect without observable toxicity to cells^26^, we demonstrated the involvement of NF-κB, p38-MAPK and ERK pathways in LL-37-mediated activation of eosinophils and BEAS-2B cells (Fig. 6). Moreover, the phosphorylation of these pivotal cell signaling molecules for the activation of these pathways was further confirmed by using quantitative flow cytometry of intracellular signaling molecules (Fig. 7). The results show that LL-37-triggered p38-MAPK/ERK phosphorylation in eosinophils and epithelial cells were further promoted by co-culture, whereas co-culture *per se* suppressed LL-37-mediated IκBα phosphorylation. Collectively, these data imply that although NF-κB pathway is indispensable for the expression of ICAM-1 and cytokines/chemokines (Fig. 6), it is not responsible for co-culture-amplified activation of cells under LL-37 stimulation (Fig. 7A). Moreover, LL-37-triggered and co-culture-amplified p38-MAPK/ERK signal may be able to compensate for the dampened NF-κB signal for optimal cell activations (Fig. 7A–C).

Our *in vivo* experiments using the OVA-induced mouse model of allergic airway inflammation indicated that antimicrobial peptide mCRAMP further enhanced key features of allergic asthma, including AHR and airway inflammation. The intensity of AHR was measured by using whole body plethysmography, while airway inflammation was assessed by inflammatory cell infiltration, mucus secretion, allergen-specific IgE production and cytokine expression. One novel aspect of the present *in vivo* study is that mCRAMP was intranasally administered together with OVA during the challenge stage of the asthmatic model, mimicking the increased airway expression of LL-37 induced by acute bacterial/viral infections during asthma exacerbations. In these experiments, enhanced severity of AHR and airway inflammation was observed when OVA-sensitized mice were intranasally challenged with a combination of OVA and mCRAMP (Fig. 8). It is worth noticing that mCRAMP alone triggered neither AHR nor airway inflammation, unless it was administered together with allergen OVA during the challenge stage (Fig. 8). The rapid IgE production might be the result of enhanced antigen presentation, memory T cell activation and increased IL-4 level triggered by LL-37-induced cytokines and chemokines (such as IL-6, KC, etc.). Therefore, our finding regarding the mCRAMP-aggravated allergic airway inflammation in mice should imply the potential pro-inflammatory role of LL-37 in infection-induced asthma exacerbation in patients.

In conclusion, using asthmatic mouse model and *ex vivo* co-culture systems, this study demonstrated a novel LL-37-stimulated pathway that induces the activation of eosinophils by interacting with bronchial epithelial cells. Taken together with previous publications showing a key role of LL-37 in immunomodulation, our findings elucidated a mechanism by which LL-37 could induce the accentuation of asthma through P2X7/EGFR and innate immunity. In addition, our results on the vicious effects of LL-37 on allergic asthma should also shed light on the feasibility of implementing therapeutic LL-37 blockage for alleviating asthma exacerbation.

## Materials and Methods

### Blood buffy coat

Fresh human buffy coat was obtained from healthy volunteers of Hong Kong Red Cross Blood Transfusion Service. Primary human eosinophils were purified from eosinophil-rich granulocyte fraction of buffy coat within 24 h after blood donation. The experimental procedure using human eosinophils purified from buffy coats was approved by Clinical Research Ethics Committee of The Chinese University of Hong Kong-New Territories East Cluster Hospitals according to the 1964 Declaration of Helsinki and its later amendments and informed written consent was obtained from all subjects.

### Mice

Inbred female BALB/c mice (8-week-old and 20 g weight) were purchased from The Laboratory Animal Services Centre, The Chinese University of Hong Kong, Hong Kong. All animal experiments were approved by the Animal Experimentation Ethics Committee of The Chinese University of Hong Kong. All experiments on animals were performed in accordance with the relevant guidelines and regulations outlined in the Animal Experimentation Ethics Committee Guide for the Care and Use of Laboratory Animal, The Chinese University of Hong Kong.

### Reagents

Synthetic human antimicrobial peptide LL-37, WRW4, KN-62, AG1478 and AG825 were purchased from Tocris Bioscience (Avonmouth, Bristol, United Kingdom). Synthetic mCRAMP was bought from AnaSpec EGT Group (Fremont, CA, USA). SB203580, BAY11-7082, U0126, LY294002 and SP600125 were obtained from Calbiochem, Merck Millipore (Darmstadt, Germany).

### Isolation of human eosinophils from buffy coat

Fresh human blood buffy coat was firstly diluted 1:2 with PBS and centrifuged using the 1.082 g/ml isotonic Percoll solution (Amersham and Pharmacia Biotech, Uppsala, Sweden) for 23 min at 900 g. To isolate eosinophils, the granulocyte fraction was collected after brief RBC lysis with water. Cells were then incubated with the anti-CD16 antibody-coated magnetic beads (Miltenyi Biotec, Bergisch Gladbach, Germany) at 4 °C for 40 min, followed by the depletion of CD16-positive cells by loading the cells onto a LS+ column (Miltenyi Biotec) within a magnetic field. The drop-through fraction containing enriched eosinophils were collected, which had a purity of at least 99% that was assessed by Hemacolor rapid blood smear stain (E Merck Diagnostica, Darmstadt, Germany). Less than 10% of the enriched eosinophils were apoptotic as assessed by Annexin V/PI staining (BD Biosciences, San Jose, CA, USA).

### Co-culture of human eosinophils with BEAS-2B cells

The human bronchial epithelial cell line BEAS-2B cells were obtained from the American Type Culture Collections (Manassas, VA, USA) and maintained in Dulbecco’s Modified Eagle Medium Nutrient Mixture F-12 medium (Gibco Invitrogen Corp, Carlsbad, CA, USA) supplemented with 10% Fetal Bovine Serum (FBS, Gibco Invitrogen Corp). Primary human bronchial epithelial cells were from Cell Applications Inc., San Diego, CA, USA and maintained with LHC-8 medium (Thermo Fisher Scientific, Rockford, IL, USA). For co-culture, the medium was changed to RPMI1640 supplemented with 10% FBS (total volume: 500 µl in 24-well cell culture plate), and 3 × 10^5^ eosinophils and 1 × 10^5^ BEAS-2B cells were co-cultured with or without antimicrobial peptide LL-37 for 20 h. For control, either 3 × 10^5^ eosinophils or 1 × 10^5^ BEAS-2B were also cultured in 500 µl RPM1640/FBS in separated wells. Except for specific indication, the concentrations of LL-37 used in the experiments were 10 µg/ml.

### Co-culture of fixed eosinophils with bronchial epithelial cells

Eosinophils or BEAS-2B cells were fixed with 1% formaldehyde to prevent the release of cytokines and chemokines, while maintaining the integrity of cell membrane. Cells were washed four times with large volume of PBS, and fixed or unfixed cells were co-cultured and stimulated with LL-37.

### Co-culture of separated eosinophils with bronchial epithelial cells using transwell inserts

To separate eosinophils and BEAS-2B cells in co-culture, 0.4 μm pore size transwell inserts (BD Biosciences) were used. Eosinophils and BEAS-2B cells were cultured together with transwell insertion, and eosinophils and BEAS-2B cells were respectively seeded in upper and lower compartment.

### Flow cytometry

To measure the surface expression of ICAM-1 and CD18 on human eosinophils and bronchial epithelial cells, cells were collected by trypsinizing with 0.25% trypsin-EDTA (Gibco Invitrogen Corp), washed with ice-cold PBS, followed by staining with FITC-conjugated mouse anti-human ICAM-1 antibody (BD Biosciences), FITC-conjugated mouse anti-human CD18 antibody (BD Biosciences) or FITC-conjugated mouse IgG1 isotypic antibody (BD Biosciences) at 4 °C for 30 minutes in the dark. After brief washing with PBS, cells were analyzed with a FACSCalibur flow cytometer (BD Biosciences).

To measure the intracellular expression of cytokines/chemokine, eosinophils and BEAS-2B cells were co-cultured and stimulated with LL-37 for 16 h, followed by the addition with 10 μg/ml Brefeldin A (Biolegend, San Diego, CA, USA) and incubated for further 4 hours. Cells were collected by digesting with 0.25% trypsin-EDTA (Gibco Invitrogen Corp) prior to fixation with a fixation buffer (Biolegend). After washing 3 times with Intracellular Staining Permeabilization Wash Buffer (Biolegend), cells were stained with biotinylated mouse anti-human IL-6, CXCL8 and CCL4 antibodies (Biolegend) or biotinylated isotypic antibodies (Biolegend). After brief washing with Intracellular Staining Permeabilization Wash Buffer, cells were incubated with FITC-conjugated streptavidin (Biolegend) for another 30 minutes in the dark before flow cytometric analysis.

Flow cytometry results were analyzed using the FCS Express software version 4.0 (De Novo Software, Los Angeles, CA, USA).

### Determination of cytokine and chemokine concentrations

Concentrations of human IL-6, human CXCL8, human CCL4, murine IL-4 and murine OVA-specific IgE were measured by using specific ELISA kits bought from Biolegend. Murine KC was quantitated using mouse chemokine Bio-Plex assay kit (Millipore, Billerica, MA, USA) on the Bio-Plex 200 system (Bio-Rad Laboratories, Hercules, CA, USA).

### Selective inhibition of FPR2, P2X7R, EGFR and ERBb2

For selective inhibition of potential receptors for LL-37-induced activation of cells, eosinophils in co-culture with BEAS-2B cells were pretreated with 5 μM WRW4 (FPR2 antagonist), 5 μM KN-62 (P2X7R antagonist), 1 μM AG1478 (EGFR antagonist), or 1 μM AG825 (ERBb2 antagonist) 1 hour before the stimulation of LL-37, and cells were cultured for further 20 hours before the analysis of the expression of adhesion molecules and cytokines/chemokines.

### Selective inhibition of intracellular signal transduction pathways

For selective inhibition of intracellular signal transduction pathways, small chemical molecule inhibitors of p38-MAPK, NF-κB and ERK pathways were used accordingly^26^. After 1 h priming with 7.5 μM SB203580 (p38-MAPK inhibitor), 1 μM BAY11-7082 (NF-κB inhibitor), 10 μM U0126 (ERK inhibitor), 5 μM LY294002 (PI3K/AKT inhibitor) or 3 μM SP600125 (JNK inhibitor), LL-37 were added to eosinophil-BEAS-2B cells co-culture and cells were cultured for further 20 h.

### Measurement of phosphorylated and total signaling molecules

For the detection of intracellular phosphorylated signaling molecules, LL-37 stimulated eosinophils and BEAS-2B cells were fixed with a fixation buffer (Biolegend) for 15 minutes at room temperature. After centrifugation, cells were permeabilized with BD Phosflow Perm Buffer III (BD Bioscience) for 30 minutes on ice. After washing 3 times with Intracellular Staining Permeabilization Wash Buffer (Biolegend), cells were stained with PE-conjugated anti-human/mouse phosphor-p38 MAPK (pT180/pY182) antibody (BD Bioscience), PE-conjugated anti-human/mouse phosphor-ERK1/2 (pT202/pY204) antibody (BD Bioscience), eFluor660-conjugated anti-human/mouse phosphor-IκBa (S32/S36) antibody (eBioscience, San Diego, CA, USA) or PE/eFluor660-conjugated isotypic antibodies (eBioscience). After brief washing with PBS, cells were subjected to flow cytometric analysis.

### Mice model for allergic airway inflammation

Murine model of allergic airway inflammation was established as previously described^38^. Briefly, on Day 0 and Day 7, mice were sensitized by intra-peritoneal injection of 50 μg OVA absorbed to alum adjuvant (Thermo Fisher Scientific). The sensitized mice were challenged once a day with PBS, 10 μg mCRAMP, 20 μg OVA, or 20 μg OVA plus 10 μg mCRAMP on Day 21, 22 and 23. Mice were subjected to analysis on Day 24. By using the whole body plethysmograph systems (Buxco-Force Pulmonary Maneuvers, Buxco Research Systems, Wilmington, NC, USA), the Penh values representing airway hyperresponsiveness were recorded by exposing mice with either water or increasing concentrations of methacholine. After sacrifice, sera were collected for the measurement of concentrations of OVA-specific IgE. Lungs were removed and homogenized in 1 ml of PBS. Levels of IL-4 and KC in the lung homogenates were then analyzed. The lung tissues were also collected from sacrificed mice for subsequent histological analysis.

### Hematoxylin and eosin (H&E) and Periodic acid–Schiff (PAS) staining

H&E and PAS staining were performed as previously described^39^. Briefly, excised lungs were fixed at 4 °C with 8% formalin for 72 hours, followed by PBS rinsing, dehydrating and paraffin-embedding. Then 4 μm sections were stained with either H&E staining kit (Beyotime Co., Shanghai, China) for the assessment of general morphology and eosinophil infiltration, or PAS staining kit (Sigma-Aldrich Corp, St. Louis, MO, USA) for the assessment of mucin producing goblet cells.

### Statistical analysis

SPSS 13.0 software (SPSS Inc, Chicago, IL, USA) were used for analyzing the data obtained. Differences among groups were assessed by One-way ANOVA. Significance was denoted by P < 0.05.
